# Supplementary material for: Comparative multiomics analysis of cell physiological state after culture in a basket bioreactor
Source: Sci Rep. 2022 Nov 23;12:20161. doi: 10.1038/s41598-022-24687-4 (PMC9686226; doi:10.1038/s41598-022-24687-4)
Supplement: Supplementary file 1 — Supplementary Information 1. [file 41598_2022_24687_MOESM1_ESM.zip › raw data/Proteomic raw data/5.GOenrichment/EG.vs.CG/ALL/EG.vs.CG.GO_Enrich.barplot.pdf]

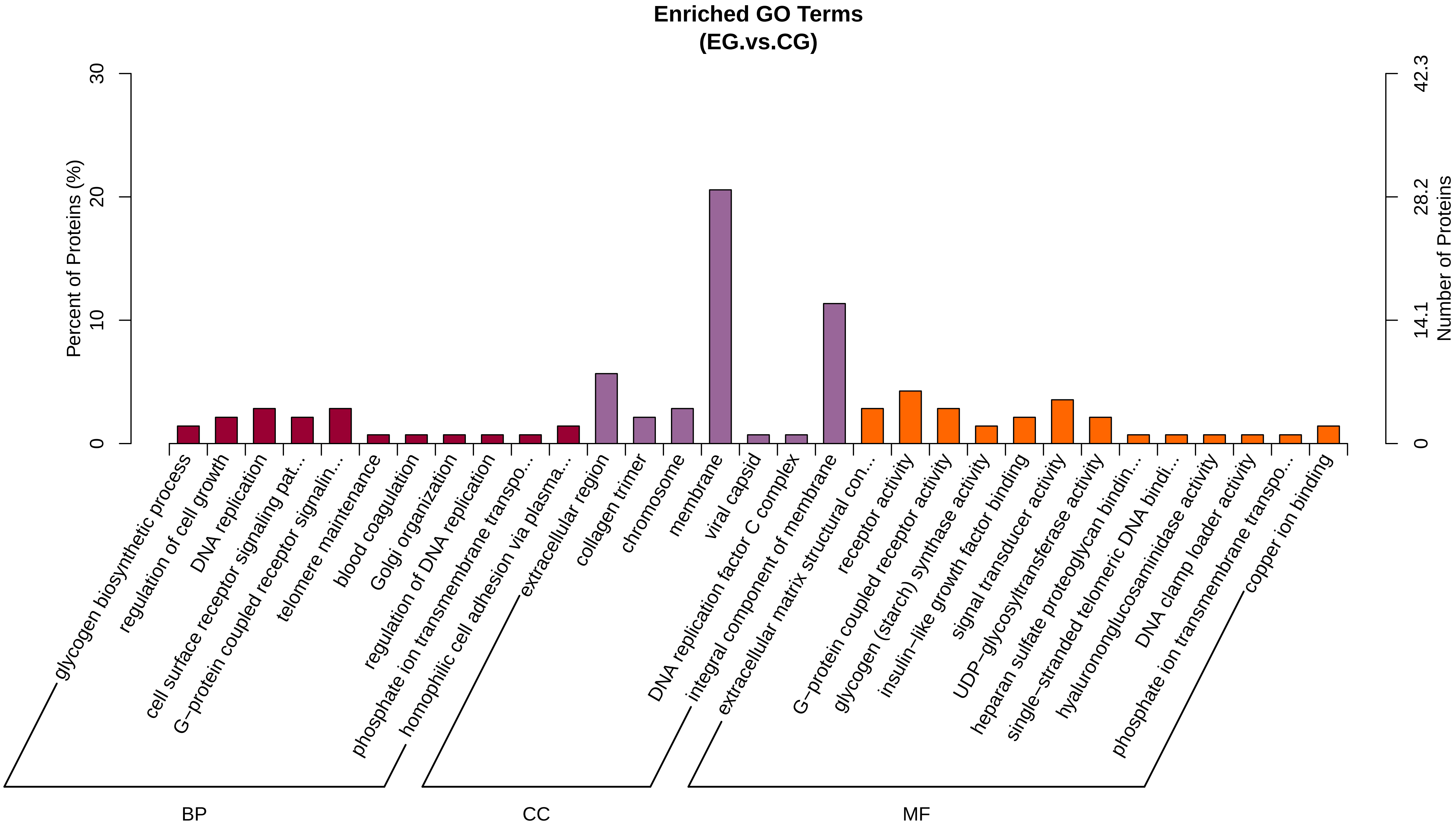

Enriched GO Terms
(EG.vs.CG)
Percent of Proteins (%)
Number of Proteins
0
10
20
30
0
14.1
28.2
42.3
glycogen biosynthetic process
regulation of cell growth
DNA replication
cell surface receptor signaling pat...
G−protein coupled receptor signalin...
telomere maintenance
blood coagulation
Golgi organization
regulation of DNA replication
phosphate ion transmembrane transpo...
homophilic cell adhesion via plasma...
extracellular region
collagen trimer
chromosome
membrane
viral capsid
DNA replication factor C complex
integral component of membrane
extracellular matrix structural con...
receptor activity
G−protein coupled receptor activity
glycogen (starch) synthase activity
insulin−like growth factor binding
signal transducer activity
UDP−glycosyltransferase activity
heparan sulfate proteoglycan bindin...
single−stranded telomeric DNA bindi...
hyaluronoglucosaminidase activity
DNA clamp loader activity
phosphate ion transmembrane transpo...
copper ion binding
BP
CC
MF
